# Quantitative CT Perfusion as a prognostic biomarker for chemotherapy response in patients with pancreatic ductal adenocarcinoma

**DOI:** 10.1007/s00261-026-05420-5

**Published:** 2026-04-10

**Authors:** Tom Perik, Geke Litjens, Natalia Alves, Ewoud J. Smit, Martijn W. J. Stommel, Erwin van Geenen, Henkjan Huisman, John Hermans

**Affiliations:** 1 Department of Medical Imaging, Radboud University Nijmegen Medical Centre, Nijmegen, Netherlands; 2 Department of Gastroenterology and Hepatology, Isala, Zwolle, Netherlands; 3 Department of Surgery, Radboud University Nijmegen Medical Centre, Nijmegen, Netherlands; 4 Department of Gastroenterology and Hepatology, Radboud University Nijmegen Medical Centre, Nijmegen, Netherlands

**Keywords:** CT perfusion, Pancreatic cancer, Treatment response, RECIST, Artificial intelligence, Biomarker

## Abstract

**Background:**

Response evaluation of pancreatic ductal adenocarcinoma (PDAC) with routine contrast-enhanced CT (CECT) using RECIST is currently inadequate for identification of patients benefiting from chemotherapy treatment, as the majority of patients show stable disease. This might lead to inappropriate treatment and potentially diminishes patients' quality of life. Recent developments in CT perfusion (CTP) show its potential as a biomarker to evaluate therapy response in PDAC.

**Purpose:**

To investigate quantitative CT Perfusion as a prognostic biomarker during chemotherapy treatment in patients with PDAC.

**Materials and methods:**

This prospective cohort trial included patients between January 2018 and December 2022 with biopsy-proven PDAC of all stages who underwent CT Perfusion before (i.e. baseline) and after three months of chemotherapy treatment. A previously developed AI-assisted CTP analysis method provided automatic segmentations of tumor and pancreas parenchyma. A trilinear non-parametric CTP contrast-enhancement model described the upslope and the peak of the time intensity curve for both tumor and parenchyma. Perfusion features of baseline and follow-up scans were compared to calculate the percentual difference. The primary endpoint was the association between quantitative CTP parameters and overall survival (OS). Secondary endpoints included comparison with RECIST 1.1 and CA 19–9 response criteria for prognostic stratification. Statistical analysis included Kaplan–Meier curves and log-rank test.

**Results:**

A total of 25 patients were included. Treatment response based on RECIST 1.1 criteria showed two cases with progressive and 23 with stable disease. Patients with increased peak enhancement after chemotherapy demonstrated significantly longer survival compared to those with decreased enhancement (p = 0.02). In contrast, CA19-9 response (≥ 30% decrease) did not significantly differentiate survival (758 days vs 371 days, p = 0.27). Furthermore, CTP found more patients with a higher chance of long survival compared to RECIST (60% vs 25% survival in 24 months). In the subgroup with RECIST stable disease CTP still finds patients with a significantly longer survival (p = 0.02). Combined analysis showed that patients with both CTP increase and CA19-9 response had the best median survival at 758 days (IQR: 561–895).

**Conclusion:**

Our study demonstrates that quantitative CTP is associated with better identification of long-term survivors than RECIST, based on increased peak enhancement after chemotherapy. Quantitative CTP may serve as a valuable complement to current treatment assessment methods in patients with PDAC.

## Introduction

Pancreatic ductal adenocarcinoma (PDAC) is the third leading cause of cancer-related death in the USA, with increasing incidence globally. Prognosis remains poor with a five-year survival of 13% [1].

Systemic chemotherapy is the standard of care for patients with non-resectable pancreatic ductal adenocarcinoma (PDAC) [2, 3]. Response rates remain low, particularly in locally advanced disease where only 12–15% of patients achieve objective response [4]. Given these limited response rates and significant treatment toxicity, accurate identification of patients likely to benefit from continued treatment is crucial. Early identification of non-responders could spare patients unnecessary toxicity from regimens such as FOLFIRINOX and preserve quality of life.

The current standard for treatment response assessment relies on Response Evaluation Criteria in Solid Tumors (RECIST 1.1) measurements on contrast-enhanced CT (CECT) [5]. However, in PDAC, these size-based criteria correlate poorly with histologic response and overall survival, limiting their clinical utility [6–9].

Another challenge with RECIST criteria is that 67% of PDAC patients demonstrate stable disease (SD) on initial post-treatment CECT, despite considerable heterogeneity in survival outcomes [10]. Current clinical guidelines recommend continuing chemotherapy for patients with SD until radiologic progression, potentially subjecting a significant proportion of patients to prolonged treatment with limited benefit and unnecessary toxicity [11].

These limitations highlight that RECIST-based evaluation on routine CECT is suboptimal for treatment assessment and therapy continuation decisions in PDAC. While serum carbohydrate antigen 19–9 (CA 19–9) is used as a complementary biomarker, it has limitations including false negatives in Lewis-negative patients and elevations due to biliary obstruction [12]. Although pathological response assessment provides valuable prognostic information it is only available in the minority of patients who undergo resection [13].

A reliable imaging biomarker that can evaluate treatment response is urgently needed for patients with PDAC. Further stratification to distinguish subgroups within RECIST stable disease could significantly improve selection of patients who would potentially benefit most from continued treatment versus those who should be spared further toxicity.

With dynamic contrast-enhanced CT (DCE-CT) or CT Perfusion (CTP) it is possible not only to assess anatomic-morphologic but also functional characteristics of the tumor. Studies on CTP as a treatment evaluation technique have shown promising results in lung, head and neck, and rectal cancer, particularly for evaluating treatment response and predicting survival [14, 15]. In three studies CTP has shown potential benefit in patients with PDAC to assess response to neoadjuvant therapy [16–18].

Despite the potential of CTP, the clinical adoption remains low due to complex workflow requirements. Current perfusion software needs manual annotations to create regions of interest, which is time-consuming and user-dependent, hampering its practical integration into clinical routine [19]. Therefore, we developed a novel method for automatic quantification of tumor perfusion with CTP using automated AI segmentations [20]. We hypothesized that our method of automated quantitative CTP could serve as an additional biomarker for assessing treatment response to chemotherapy.

In this prospective cohort study, we investigate the use of CTP for assessing response to chemotherapy in patients with PDAC. Furthermore, we investigate the ability of CTP to further stratify patients with RECIST stable disease into prognostic subgroups.

## Materials & methods

### Patients

Institutional review board approval was obtained, and all patients gave written informed consent. This is a prospective substudy of the DIAPANC study, registered in the US National Library of Medicine registry (https://clinicaltrials.gov/) under identifier: NCT05669287.

Between January 2018 and September 2022, consecutive patients with a clinical suspicion of pancreatic cancer, as determined in the multidisciplinary tumor board were prospectively enrolled. Inclusion criteria required patients to undergo CT perfusion imaging before and approximately three months after initiation of chemotherapy treatment. Exclusion criteria were age below 18 years, previous treatment for pancreatic cancer, concomitant malignancies, contraindications to CECT (i.e. untreatable contrast allergy, severe renal function impairment), and insufficient command of the Dutch language. For the final analysis only patients with histopathologic proof of PDAC, obtained by fine needle aspiration/biopsy (FNA/FNB) or surgical resection, were included. The image quality needed to be sufficient to analyze the time-intensity curve.

Relevant clinical data included demographics (age, sex), tumor size and location, TNM stage, CA 19–9 levels, treatment details (resection, chemotherapy), histopathology and survival. Overall survival was calculated from the date of study enrolment, which occurred at initial hospital presentation prior to baseline imaging. Data was retrieved from electronic medical records.

### Image acquisition

All CT examinations were performed on a 160 mm wide-detector 320-row scanner (Canon Aquilion ONE Genesis Edition, Canon Medical Systems Corporation). The contrast-agent (Iomeprol 400 mg/mL, Bracco) was body-weight based (1.3 mg I/kg) and intravenously administered with a fixed duration of injection time of 5 s. A 5 mL saline bolus chase was injected directly after the contrast agent injection.

**Fig. 1:**
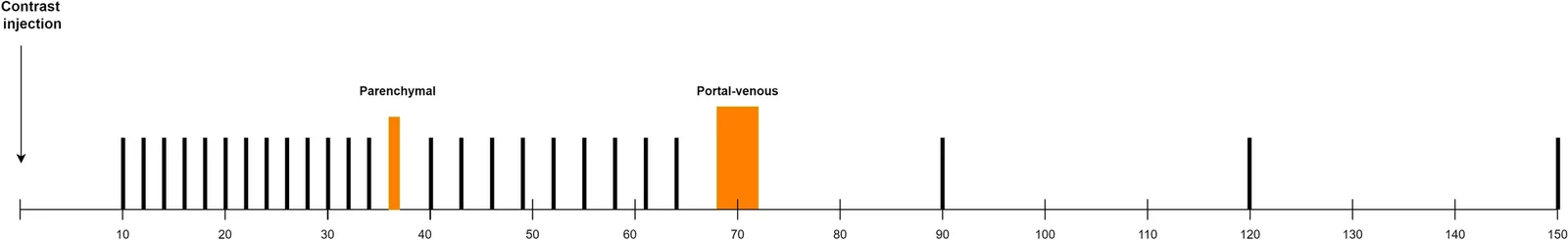
The interleaved CT perfusion protocol integrates dynamic perfusion scans (black bars) with diagnostic phases (orange bars): non-contrast, parenchymal, and portal-venous

A test bolus of 15 mL was used to determine the time of contrast arrival in the aorta at the level of the pancreas. Based on the test bolus, the perfusion series started 2 s before the measured contrast arrival time in the aorta, consisting of 13 acquisitions with 2-s intervals, followed by a diagnostic volume scan in the parenchymal phase around 35 s. Nine subsequent dynamic acquisitions were obtained at 3-s intervals. At 70 s a portal-venous diagnostic helical scan of the thorax and abdomen was performed, followed by three dynamic acquisitions at 90, 120, and 150 s. Only the upper abdomen was scanned in the perfusion scans and in the diagnostic parenchymal phase. The complete scan protocol is denoted in Fig. 1 and was described in a previous study [20].

Post-treatment CT perfusion follow-up was performed at three months after baseline examination, coinciding with standard first treatment response evaluation.

### CTP AI biomarker

A previously developed biomarker comprised a pipeline of three modules to transform a CTP image into tumor perfusion features [20].

Briefly this involved: first, non-rigid image registration using Body Registration software (Canon Medical Systems); second, a fully automated deep learning model (nnUnet framework) for 3D segmentation of tumor and pancreas parenchyma [21]. This model, trained on portal-venous phase scans with histopathological proven PDAC, created segmentations that were applied to all timepoints to extract time intensity curves (TIC). All segmentations were validated by by an abdominal radiologist (J.H.) with over 25 years of experience in pancreatic imaging, who made necessary adjustments and excluded vessels and stents to reduce artifacts. Third, an automated trilinear curve fit based on a pharmacokinetic analysis package identified the maximum derivative in the TIC, then centered the trilinear fit around this point using 10 surrounding timepoints. This approach separated the curve into non-enhancement, upslope, and washout phases, enabling calculation of key perfusion parameters: slope and peak enhancement.

**Fig. 2:**
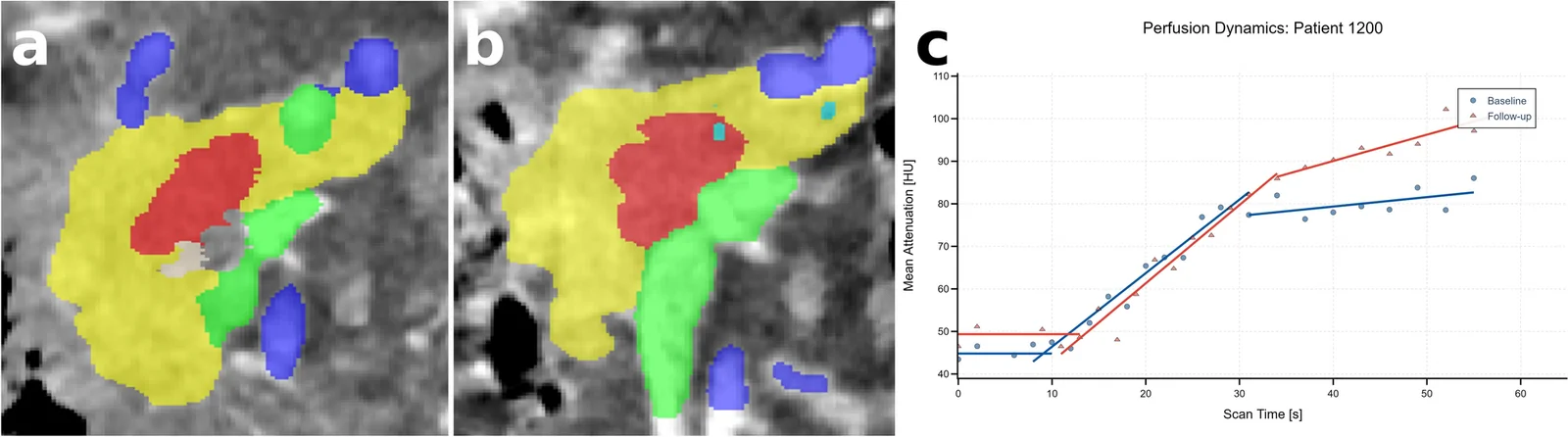
Representative CT perfusion images and analysis. **A** Baseline CTP image with AI-generated tumor segmentation (red),pancreas parenchyma segmentation (yellow), arteries (green) and vessels (blue). **B** Follow-up CTP at 3 months with corresponding segmentations. **C** Time-intensity curves demonstrating increased peak enhancement at follow-up (orange) compared to baseline (blue)

For follow-up analysis we calculated the percentual difference between peak enhancement at pre-treatment scan compared to peak enhancement in the post-treatment scan, this method was also used for the upslope. Representative examples of the automated tumor segmentation and time-intensity curves are shown in Fig. 2.

### Response to chemotherapy

Treatment response was based on RECIST 1.1 and CA19-9 at baseline and 3-months follow-up coinciding with standard first treatment response evaluation.

CA19-9 response was defined as > 30% decrease from baseline value, based on a large retrospective study ([22]. In case of resection, pathology response was based on the Evans criteria, and classified as almost complete regression, partial regression or no regression [23].

Resectability was determined using Dutch Pancreatic Cancer Group (DPCG) criteria classifying tumors as resectable, borderline resectable, or unresectable [24].

### Statistical analysis

Descriptive statistics were used to summarize the clinical characteristics of the study population, with continuous variables expressed as median (interquartile range) and categorical variables as frequencies (percentages). The Mann–Whitney U test was used to compare perfusion features. The chi-square test was used to analyze categorical variables.

For the quantitative CTP analysis, the percentual change in tumor peak enhancement was calculated (post- vs. pre-treatment). A threshold was identified by selecting the value that maximized survival separation, stratifying patients into groups with increased versus decreased enhancement. To prevent overfitting and ensure clinical validity, each resulting subgroup was required to contain a minimum of 25% of the cohort.

Overall survival (OS) was assessed from study inclusion to death or last date of contact using Kaplan–Meier method and compared between groups with log-rank test. P < 0.05 was considered statistically significant.

## Results

### Patient characteristics

**Fig. 3:**
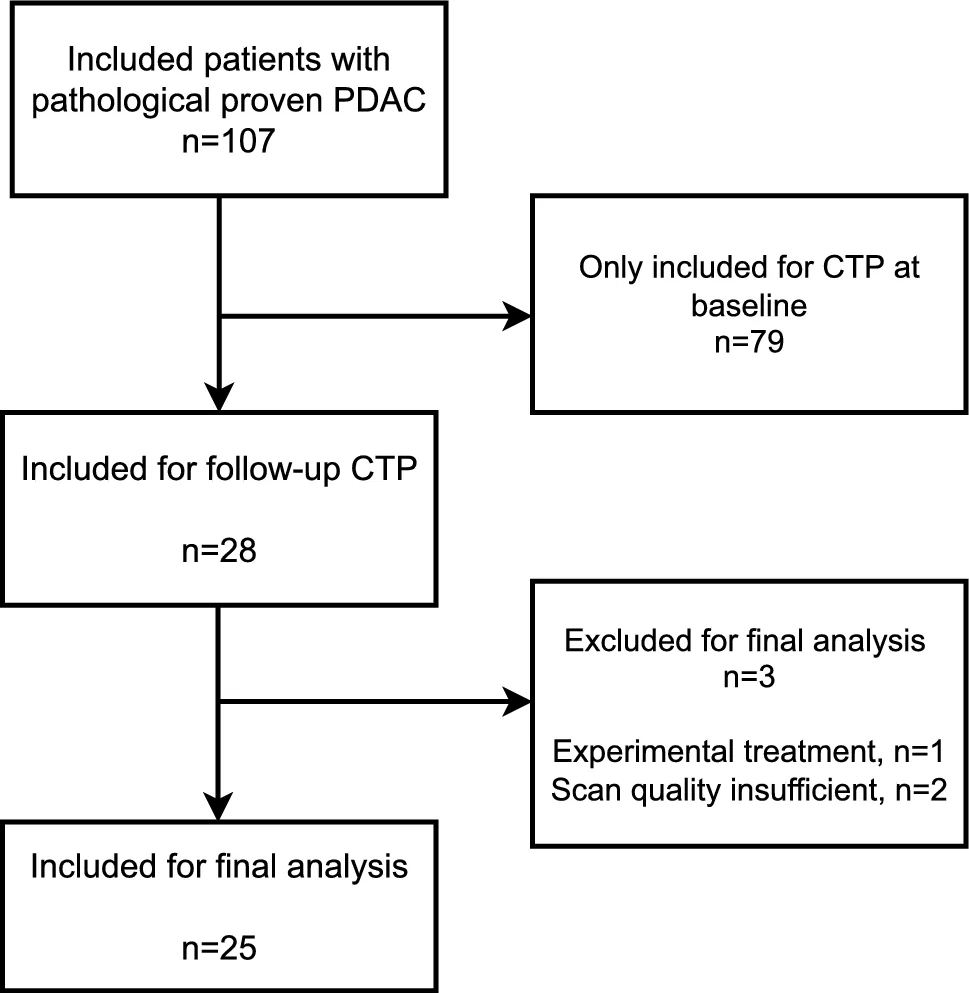
Flowchart study inclusion

Of the initial 107 patients with stage I–IV histopathological confirmed PDAC, 28 underwent a follow-up CTP scan after three months of systemic chemotherapy. Three patients were excluded due to poor scan quality (n = 2) or experimental treatment (n = 1), resulting in a cohort of 25 patients (mean age 67, 44% male) for final analysis (Fig. 3). Clinical characteristics are summarized in Table 1, with follow-up data in Table 2.

**Table 1:** Baseline characteristics of the study cohort

Variable	
Age (year)	67 (55–70)
Sex	
Male	11 (44%)
Female	14 (56%)
Tumor size pre-treatment (mm)	35 (30–42)
cTNM	
II	12 (48%)
III	8 (32%)
IV	5 (20%)
CA19-9 level at baseline (U/mL)	334 (2–7500)
Tumor location	
Head	15 (60%)
Body/Tail	10 (40%)
Type of chemotherapy	
FOLFIRINOX	23 (92%)
Gemcitabine/nab-paclitaxel	2 (8%)

Unless otherwise specified, data are medians with range in parentheses or numbers of participants with percentages in parentheses

### Treatment assessment

**Fig. 4:**
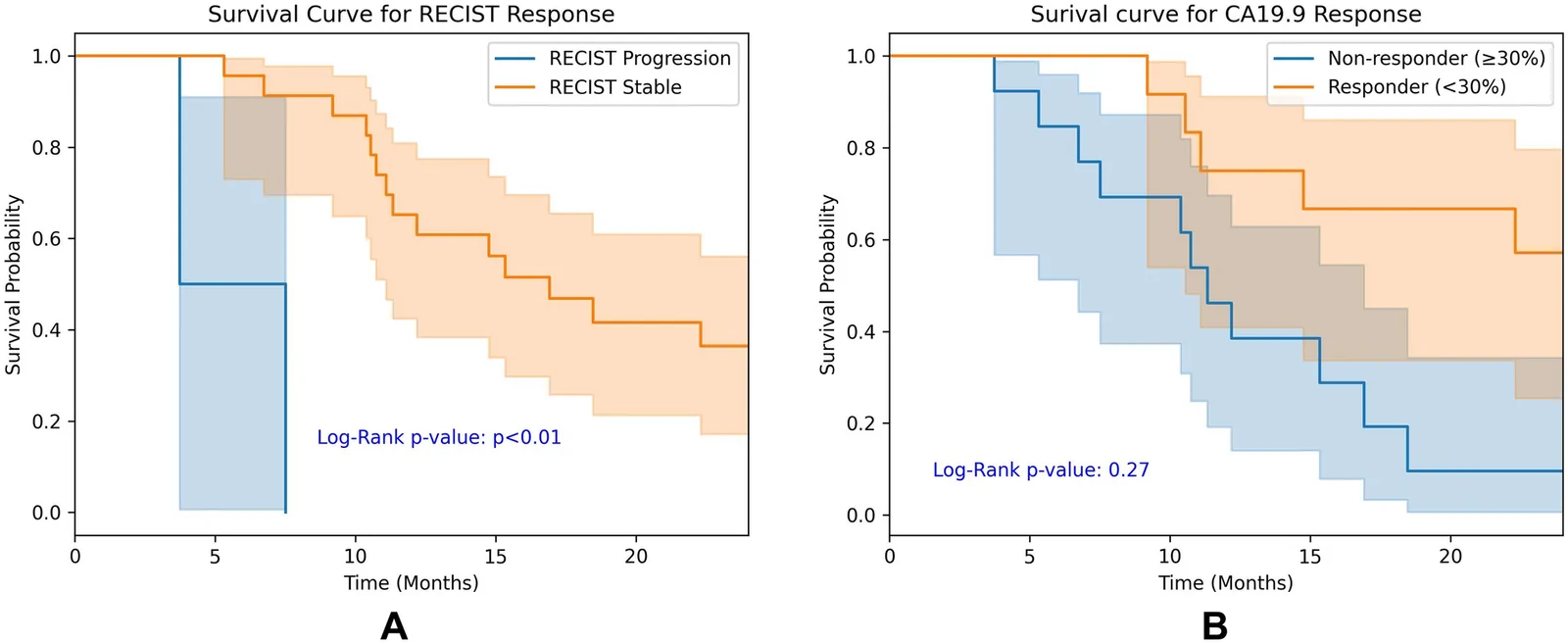
Kaplan–Meier survival curves based on (**A**) RECIST 1.1 and (**B**) CA19-9 response evaluation

RECIST 1.1 assessment showed predominantly stable disease (n = 23, 92%) with two cases of progressive disease (8%). No partial or complete responses were observed. Patients with stable disease showed significantly longer survival compared to those with progressive disease (p < 0.001, Fig. 4a).

CA19-9 response analysis defined as ≥ 30% decrease from baseline, identified 12 patients (48%) as responders and 7 patients (28%) as non-responders. Six patients (24%) maintained consistently non-elevated CA 19–9 levels (< 37 U/mL) throughout treatment. Survival analysis stratified according to biochemical response showed no significant difference (p = 0.27, Fig. 4b).

### Survival analysis using AI-assisted CTP

**Fig. 5:**
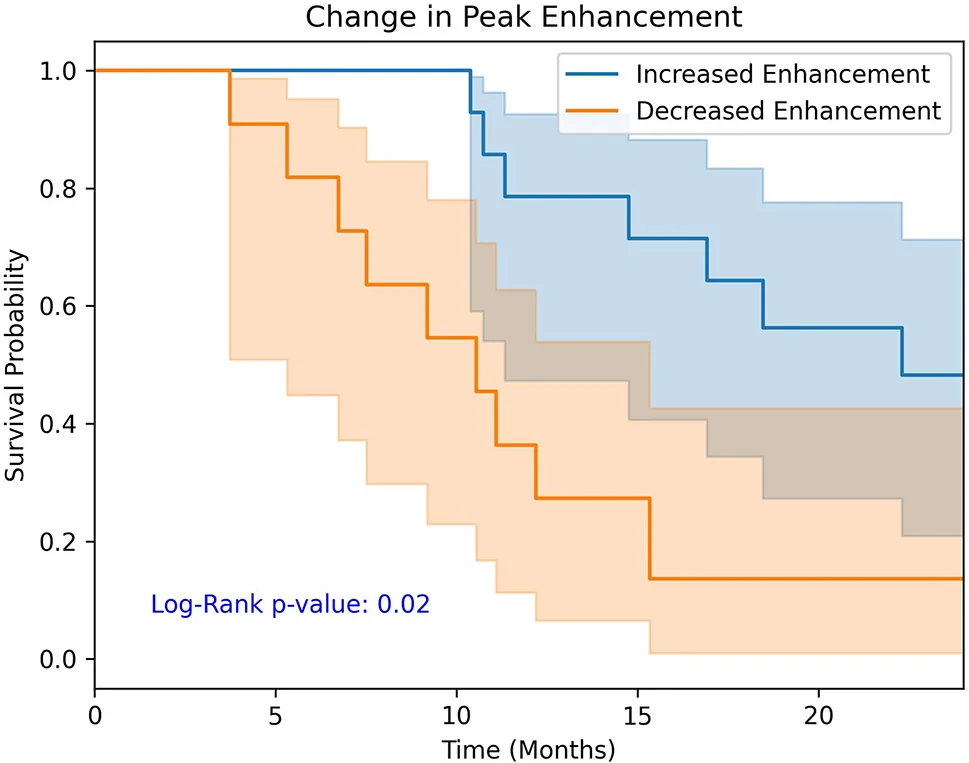
Kaplan Meier survival curve shows significantly longer survival for patients with an increased peak enhancement after chemotherapy (p = 0.02)

Quantitative CTP analysis identified two distinct groups based on peak enhancement. Patients with increased peak enhancement (n = 14/25) had longer median survival compared to those with decreased enhancement (22.3 months (IQR: 14.8–29.4) versus 12.2 months (IQR: 9.2–18.5), p = 0.042, Fig. 5). The 12-month survival rates were 78% for the increased peak enhancement group versus 36% for the decreased peak enhancement group.

**Table 2:** Follow-up characteristics of the study cohort

Variable	
Tumor size post-treatment (mm)	31 (18–84)
Scan interval weeks	13.8 (8–22)
RECIST1.1	
Partial response	0 (0%)
Stable disease	23 (92%)
Progressive disease	2 (8%)
CA19-9 level at 3 months (U/mL)	171 (2–7900)
CA19-9 response	
Response (≥ 30% decrease)	12 (48%)
Non-responder (< 30% decrease)	7 (28%)
Consistently non-elevated (< 37 U/mL)	6 (24%)
Median overall survival (months)	14 (10–24)
Resected tumors	9 (36%)
Pathology Response (Evans)	
Almost complete regression	1 (11%)
Partial regression	7 (78%)
No regression	1 (11%)

Unless otherwise specified, data are medians with range in parentheses or numbers of participants with percentages in parentheses

An increased post-treatment tumor upslope on the time intensity curve (TIC) was associated with a trend toward longer survival, but this was not statistically significant (p = 0.12).

### Subgroup analysis

**Fig. 6:**
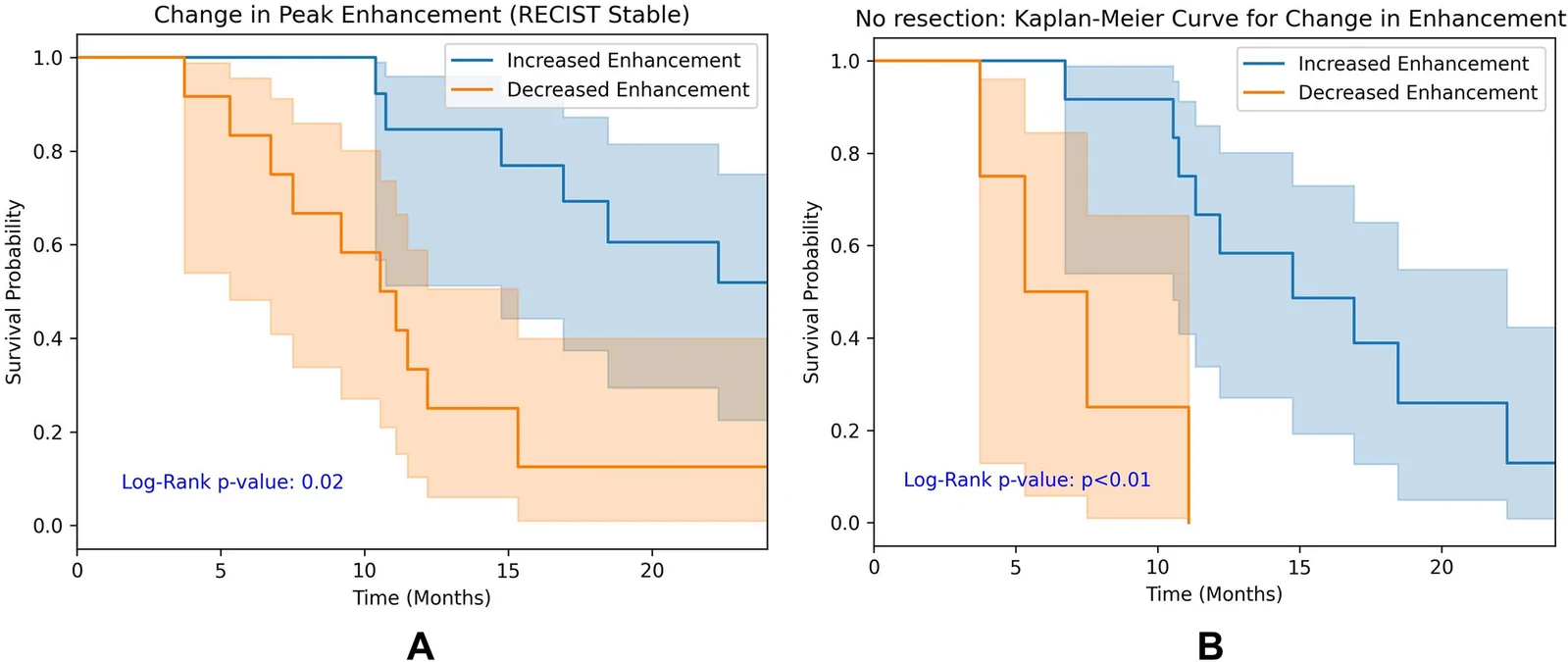
Kaplan Meier survival curves show significantly longer survival for patients with an increased peak enhancement after chemotherapy both in A) RECIST stable patients (n = 23) (p = 0.02) and B) Non-resected patients (n = 16) (p < 0.01)

Given the limited sample size, the following subgroup analyses are exploratory and hypothesis-generating. Using only patients classified as RECIST stable disease (n = 23), increased perfusion after chemotherapy on CTP was associated with a longer survival (p = 0.02), Fig. 6a.

Additionally, in the subgroup with non-resected cases (n = 16) an increased perfusion correlated with longer survival (p < 0.01), Fig. 6b.

#### Combined CTP and CA19-9

Combined analysis of CTP and CA19-9 response identified four prognostic subgroups (Table 3). Patients with both increased CTP and CA19-9 response (n = 7) demonstrated the best outcomes with median survival of 24.9 months (IQR: 18.4–29.4) and 86% 12-month survival. In contrast, patients with decreased CTP despite CA19-9 response (n = 5) had median survival of only 11.1 months, suggesting that CTP provides independent prognostic information beyond CA19-9 alone.

**Table 3:** Combined analysis of CTP and CA19-9 response for patients classified as RECIST stable

Group	N	Resected n (%)	Median Survival months (IQR)	12-month survival n (%)
CTP↑ / CA19↓	7	3 (43%)	24.9 (18.4–29.4)	6 (86%)
CTP↑ / CA19↑	5	2 (40%)	11.3 (10.7–16.9)	3 (60%)
CTP↓ / CA19↓	5	3 (60%)	11.1 (10.5–31.8)	3 (60%)
CTP↓ / CA19↑	6	1 (17%)	13.1 (6.7–15.3)	1 (17%)

*CTP↑* Increased CTP peak enhancement (post vs. pre-treatment), *CTP↓* Decreased CTP peak enhancement, *CA19↓* Biochemical Response (≥ 30 decrease from baseline), *CA19↑* Biochemical Non-response (< 30% decrease or consistently non-elevated)

## Discussion

Our study demonstrates that AI-assisted CT perfusion analysis is able to identify long-term survivors within the RECIST stable group of PDAC patients. While previous studies explored CTP parameters in PDAC, they focused on treatment response prediction rather than long-term survival outcomes [16, 17]. To our knowledge, this is the first study correlating CT perfusion biomarkers directly with overall survival in PDAC. 

CTP maintained its prognostic value across clinically relevant subgroups. Within the RECIST stable disease group, which constituted the majority of our patient cohort, quantitative CTP analysis revealed significant survival differences, indicating biological responses not detectable by size-based measurements. In non-resected patients, CTP significantly stratified overall survival, highlighting its potential value in guiding treatment decisions for patients managed with systemic therapy alone.

RECIST criteria have limitations in PDAC evaluation, where tumors frequently maintain a stable size despite treatment. While Park et al. and Hamdy et al. showed baseline perfusion parameters could predict response, our study found that post-treatment perfusion changes correlated with survival outcomes.

Assessing treatment response in pancreatic adenocarcinoma is challenging, explaining the poor correlation between RECIST response and outcomes. Radiographic imaging often fails to measure tumor shrinkage, even when treatment is effective. This is likely due to the dense stromal component remaining unchanged and the development of treatment-related fibrosis. These factors can mask actual tumor response on CT scans, limiting the effectiveness of RECIST criteria in pancreatic cancer [9, 25]. In contrast, quantitative CTP addresses this limitation by capturing functional changes in tumor vascularity and perfusion, thereby providing a biomarker for evaluating treatment efficacy in PDAC.

An earlier study used CTP in evaluating treatment response in patients with PDAC, their major finding was that higher baseline blood flow was associated with a better histopathologic response to therapy. [17] While our study did not identify pre-treatment perfusion differences, the characteristics of our dataset and the distribution of responders limited our ability to conduct a robust comparison based on pathological response. However, our results show that longitudinal CTP measurements can identify subsets of patients who may derive greater benefit from continued chemotherapy, potentially improving outcomes for patients who might otherwise be considered non-responders based on RECIST criteria alone.

To the best of our knowledge no previous study has used CTP for further stratification of patients with stable disease after initial CT. Unlike traditional anatomical biomarkers such as RECIST, which categorizes patients based on changes in tumor size, CTP offers additional information based on perfusion and vascular characteristics. Our AI-assisted approach enables objective quantification of these perfusion patterns without the variability of manual measurements. This helps to identify patients who demonstrate functional tumor response but do not show anatomical changes during treatment. Adding CTP during treatment assessment might improve patient selection by identifying patients who benefit from further chemotherapy and have a longer survival.

The use of CA 19–9 as a biomarker for PDAC treatment response also presents challenges. While we utilized a 30% decrease as a response criterion based on the literature, there remains a lack of standardized guidelines for CA 19–9 interpretation in treatment monitoring. Our analysis showed that CA 19–9 response did not correlate with survival differences (p = 0.27), highlighting the limitations of relying solely on this biomarker. The integration of CTP with both anatomical (RECIST) and biochemical (CA 19–9) markers offers a more comprehensive assessment approach. This combined strategy may better capture PDAC's complex biology and heterogeneous treatment response [26].

This study has several limitations. First, as an exploratory single-center study, our sample size is relatively small (n = 25), which limits statistical power and generalizability. These results should be considered hypothesis-generating and require validation in larger, multicenter cohorts across different CT platforms. Second, we performed only one post-treatment CTP at a three-month follow-up, preventing assessment of earlier changes or longer-term perfusion dynamics. Third, the heterogeneity of our cohort, including patients who ultimately underwent resection (n = 9) and those who did not (n = 16), creates potential confounding factors. Fourth, the timing of follow-up CTP varied, which may have influenced perfusion measurements. Standardized imaging timepoints would improve reproducibility in future studies. Fifth, the subgroup analyses (RECIST stable disease, non-resected patients) were exploratory with smaller subgroup sizes and require validation in larger cohorts. Despite these limitations, the significant survival differences observed suggest that CTP may provide meaningful prognostic information that warrants further investigation in larger, prospective studies.

Future research should focus on validating our findings in larger, multicenter cohorts. Prospective studies examining CTP at multiple timepoints during treatment could help determine optimal timing for assessment and potentially identify early perfusion changes that predict response. In the neoadjuvant setting, CTP-based response assessment may help predict which patients will achieve resectability. Additionally, correlating CTP findings with molecular markers and tumor microenvironment characteristics may provide deeper insights into the biological mechanisms underlying perfusion changes. The integration of CTP with other functional imaging modalities such as PET-CT and photon-counting CT could further enhance treatment response assessment. Finally, standardization of perfusion parameters and post-processing techniques would facilitate broader clinical implementation of CTP in PDAC management.

In conclusion, CTP provides prognostic information complementary to RECIST and CA19-9, enabling stratification within RECIST stable disease. These findings are hypothesis-generating and require validation.

## Data Availability

The datasets used and analyzed during the current study are available from the corresponding author on reasonable request.
